# Modeling Farm Equipment Vehicle crash injury severity using random parameters logit and multi-class support vector machine in a developing country

**DOI:** 10.1038/s41598-026-42267-8

**Published:** 2026-04-04

**Authors:** Ehsan Jafari Nasab, Abdoreza Sheikholeslami, Jose Manuel Vassallo, Amin Moeinaddini

**Affiliations:** 1 Department of Transportation, Faculty of Civil Engineering, Iran University of Science and Technology, Tehran, Iran; 2 Transport Research Centre – TRANSyT, Universidad Politécnica de Madrid, Madrid, Spain; 3 Key Laboratory of Road and Traffic Engineering of Ministry of Education, Tongji University, Shanghai, China

**Keywords:** Farm equipment vehicle (FEV)-involved crashes, Rural road safety, Injury severity, Heterogeneity, Random parameters ordered logit (RPOL), Multi-class support vector machine (MC-SVM), Engineering, Mathematics and computing, Risk factors

## Abstract

Farm Equipment Vehicles (FEVs) pose major safety challenges on interurban and rural roads, especially in developing countries where outdated fleets and poor infrastructure are common. Interactions among multiple risk factors often increase crash severity. This study investigates the severity of FEV-involved crashes and evaluates models for predicting accident outcomes with greater accuracy for Intelligent Transportation Systems (ITS). Two advanced methods are applied: a Random Parameters Ordered Logit (RPOL) model to capture observed and unobserved heterogeneity, and a Multi-Class Support Vector Machine (MC-SVM) to benchmark predictive performance. The RPOL results highlight key factors influencing crash severity, including overturns, time of day, road type, and involvement of other vehicles. Motorcycle involvement and nighttime crashes significantly raise fatality risks, while rear-end collisions and reversing maneuvers are linked to lower severity. Heterogeneity is observed in collisions where FEVs are at fault, particularly on straight roads. The MC-SVM model demonstrates superior predictive accuracy, achieving higher Area Under the Curve (AUC) scores compared to RPOL. Policy implications include renewing FEV fleets, restricting older vehicles, and imposing stricter controls on nighttime operations. Integrating statistical and machine learning approaches provides a robust framework for improving safety and reducing crash severity in rural transportation systems.

## Introduction

The issue of road safety in developing nations, including low- and middle-income countries (LMICs), has been of growing concern in recent years due to their higher crash and fatality rates^1–3^. On the other hand, Farm Equipment Vehicles (FEVs), including tractors, harvesters, and transport equipment, play a fundamental role in rural economies, particularly within developing countries. The integration of FEVs into regular traffic on roads presents significant safety problems, and crashes involving these vehicles represent a considerable risk to the safety of both farm laborers and other road users in rural states^4–6^. These vehicles often operate at lower speeds, lack modern safety features, and are frequently driven under suboptimal visibility or environmental conditions, making them susceptible to severe and fatal crashes^7–9^. The recent reviews found that crash risk increases when these large, slow-moving vehicles share public roads under conditions of low visibility or adverse weather^10–12^. Moreover, a Dutch study tracking decades of data showed that FEVs continue to be overrepresented in severe collisions, primarily due to factors like vehicle size, limited visibility, and lack of dedicated infrastructure separation^13^. As food production demands and rural mobility continue to grow, understanding the dynamics of FEV-related crashes becomes increasingly critical for public safety and infrastructure planning.

Globally, traffic crashes involving FEVs are a major concern, especially in rural areas of developing countries where inadequate infrastructure, limited enforcement, and outdated machinery exacerbate the risks. In Iran, farm road traffic fatalities have consistently represented a significant share of rural traffic deaths from 2019 to 2024. Despite this trend, most transportation safety research has traditionally focused on urban traffic systems and passenger vehicles, often overlooking the distinct operational environments and risk factors associated with FEV. According to the Iranian Ministry of Agriculture, the national fleet includes approximately 600,000 to 620,000 FEVs^14^. A considerable proportion of this fleet (particularly tractors and combines) is aging, with over half of the tractors having been in service for more than 13 years.

Crashes in rural areas disproportionately affect local communities. International data indicate that, when adjusted for vehicle miles traveled, fatality rates in rural crashes are twice as high as those in urban settings^15–17^. Furthermore, crash fatality rates involving FEV are nearly five times higher than those of other rural traffic incidents^15,18^. Based on the 2024 estimates, approximately 20.35 million people (22.3% of Iran’s total population of ~ 87.6 million) reside in rural areas. Of the 19,435 road traffic fatalities reported in Iran in 2024, 1,089 (5.6%) occurred on rural roads, 13,372 (69%) on interurban roads, and 4,759 (25.4%) in urban areas. Notably, many interurban routes also serve as corridors for transporting farm products, especially during peak harvest periods. The operational differences and speed mismatches between farm and standard vehicles on these roads frequently lead to severe collisions.

To explore a more accurate model in predicting the severity class of FEV-involved understand the crash severity associated with FEVs, this study employs a dual-method approach: the Random Parameters Ordered Logit (RPOL) model and the Multi-Class Support Vector Machine (MC-SVM) model. It is worth noting that the RPOL model captures both observed and unobserved heterogeneity in crash severity^19–23^, while the MC-SVM model, a robust machine learning algorithm, enhances predictive performance and identifies non-linear relationships among crash factors^24–27^. Together, these methods provide a comprehensive framework for analyzing injury severity in FEV-involved crashes. It must be noted, based on the best knowledge of the authors, that no previous study accounts for the interaction effect of factors on the severity of FEV-involved crashes (especially in the fields of developing countries).

A distinctive feature of FEV crashes is that injury severity rarely results from the isolated effect of a single factor but emerges from complex interactions among driver behavior, vehicle characteristics, road geometry, and environmental conditions. For instance, a slow-moving tractor may pose minimal risk on a curved rural road yet become extremely dangerous on a straight high-speed corridor when struck from behind, or an overturn that is survivable on flat terrain can turn fatal on a straight alignment due to higher pre-crash speeds. To the best of the authors’ knowledge, no previous study on FEV-involved crashes—particularly in developing countries—has systematically modeled two-way interaction effects or the heterogeneity in how these interactions influence severity outcomes. Ignoring such interactions can mask critical risk patterns and lead to misleading policy recommendations.

The remainder of this paper is organized as follows: Section "Literature review" reviews the relevant literature on transportation safety. Section "Methodology" outlines the methodology employed. Section "Case study" describes the case study and data sample. Section "Results" presents the empirical results and discusses the findings.

## Literature review

Research into FEV crashes on public roads reveals a complex interplay of vehicle, driver, and environmental factors contributing to injury severity. Recent advances in traffic safety modeling have increasingly adopted heterogeneous and data-driven approaches to better capture the complex factors influencing crash severity, especially in the context of specialized vehicles such as FEVs. *Peek-Asa, Sprince*^28^ found that non-farm vehicle drivers are over five times more likely to be injured in crashes involving farm equipment, especially when restraint use is absent, crashes occur in low-light conditions, or when risky behaviors such as speeding or unsafe passing are involved. *Costello, Schulman*^29^ expanded on this by identifying farm-level risk factors: farms employing non-English speaking or non-family drivers, those with younger drivers, or a history of FEV injuries are significantly more likely to be involved in crashes. *Myers, Cole*^30^ conducted a detailed analysis of tractor overturn events to determine how specific overturn characteristics influence injury severity. Using data from 410 tractor overturns collected through structured interviews in Kentucky, the study found that side overturns were significantly more common than rear overturns, and overturns on sloped terrain greatly increased the odds of serious or fatal injury. The absence of rollover protective structures (ROPS) was strongly associated with more severe outcomes. Other critical factors included the speed of operation, ground conditions, and whether the operator was thrown from the tractor. Importantly, the study emphasizes that engineering interventions—particularly universal ROPS implementation—are key to reducing injury severity in overturn incidents and improving FEV safety overall. *Gkritza, Kinzenbaw*^31^ conducted a multinomial logit analysis of crash severity, showing that crash characteristics (rear-end and single-vehicle collisions), driver factors (young drivers), vehicle attributes (older equipment), and contextual conditions (darkness, rural location, harvest season) significantly influence the likelihood of severe or fatal outcomes. Collectively, these studies highlight the need for multifaceted prevention strategies encompassing vehicle visibility enhancements, targeted education, better road infrastructure, and more informed farm management practices.

*Pinzke, Nilsson*^32^ investigated farm tractor incidents on Swedish public roads with a specific focus on age-related differences among drivers. Analyzing police-reported crash and injury data from 2003 to 2010, the study revealed that older tractor drivers (aged 65 and above) were overrepresented in single-vehicle crashes, which often involved poor maneuvering and limited situational awareness. Younger drivers (aged 15–24) were more likely to be involved in speed-related or risky behavior incidents. Injury outcomes were more severe in collisions involving larger vehicles or when vulnerable road users (e.g., cyclists, pedestrians) were struck. These findings emphasize the importance of targeted road safety campaigns and driver training programs for both older and younger tractor operators. *Harland, Greenan*^7^ explored differences in FEV crash characteristics based on rural–urban location and proximity to town centers in nine Midwestern states. Using 2005–2010 police-reported crash data, the study found that although the majority of farm equipment crashes occurred in rural areas, nearly 30% happened in urban or peri-urban areas—often within 25 miles of town centers. Urban and near-town crashes were more likely to involve non-farm vehicles and occurred more frequently at intersections, suggesting visibility and interaction complexity as critical issues. In contrast, rural crashes were more likely to result in rollovers and occur on high-speed roads. The study underscores the need to broaden the perception of FEV road crashes as not just a rural issue and calls for infrastructure improvements and awareness efforts targeting urban-adjacent areas. *Anarkooli, Hosseinpour*^33^ used a six-year dataset from Malaysia to examine injury severity outcomes in single-vehicle (SV) rollover crashes using a Random-Effects Generalized Ordered Probit (REGOP) model. The study found that several factors significantly increase the likelihood of severe or fatal outcomes (KSI), including dark, unlit conditions, rain, undulating terrain, improper overtaking, older vehicles, and high-speed zones. The presence of heavy vehicles and light trucks (SUVs, vans) was also positively associated with severity. In contrast, wider unpaved shoulders, more access points, and urban settings were associated with lower severity. The REGOP model outperformed the mixed logit model, showcasing its strength in capturing both ordinal nature and unobserved heterogeneity in crash data, making it a promising tool for injury severity modeling in rollover incidents.

*Harland, Bedford*^34^ examined the role of alcohol impairment in on-road farm equipment crashes across four Great Plains states (Iowa, Missouri, North Dakota, and South Dakota) from 2005 to 2010. Out of 1,971 crashes, 3.1% involved an alcohol-impaired driver, and these incidents were disproportionately represented in fatal crashes (17.8%) and injury crashes (5.6%). Alcohol-impaired drivers, particularly those operating non-farm vehicles, were significantly more likely to be involved in injury or fatal crashes. Multivariable logistic regression revealed that crashes involving an alcohol-impaired driver had 4.10 times higher odds of resulting in injury or fatality. The findings highlight alcohol impairment—especially among non-farm vehicle drivers—as a key public health concern in FEV traffic safety. *Scott, Hirabayashi*^18^ analyzed FEV-related crashes in rural New York State using data from the Department of Motor Vehicles (DMV) crash records between 2010 and 2012. The study found that FEV crashes had a case fatality rate nearly five times higher than non-FEV crashes. These incidents were most likely to occur during daylight hours and on straight, graded roadways. Moreover, FEV were more frequently involved in two-vehicle collisions and were more often struck by other vehicles rather than being the striking party. Injuries resulting from these crashes were disproportionately moderate to severe in nature. The findings underscore the elevated risk posed by FEVs in mixed-traffic environments and highlight the need for safety interventions such as improved visibility and public driver awareness in rural regions. *Cai, Wei*^35^ explored the injury severity of rural single-vehicle crashes under low-visibility conditions using a spatial random parameters model with crash data from Kansas. The study emphasized the importance of accounting for spatial correlation and unobserved heterogeneity to better understand contributing factors. Results showed that fog, curved roads, and early morning crashes significantly increased the likelihood of severe injuries. Furthermore, the model revealed substantial spatial variation in how crash factors influence injury severity across different regions. The findings underscore the necessity for localized countermeasures and improved warning systems in rural areas prone to low visibility, particularly during nighttime or foggy conditions. *Kim, Kim*^36^ conducted a nationwide study in South Korea using FEV insurance data to assess injury incidence rates across various machine types between 2014 and 2020. Analyzing over 338,000 insurance subscriptions and 2,061 injury cases, the study found an average injury rate of 6.1 per 1,000 machines, with higher rates associated with power carts (18.7/1,000) and power tillers (18.0/1,000). Elderly users (particularly those aged 60 +) and machines like power carts and tillers had the highest injury risks. Logistic regression showed the odds of injury were more than twice as high for these machines compared to tractors. The results stress the importance of targeted legal and technical safety measures, including driver age restrictions and machine-specific regulation, to reduce FEV-related injuries. *Ren, Xu*^37^ analyzed single-vehicle rollover crashes in California from 2013 to 2017 using a random parameters logit model with heterogeneity in means and variances. Injury severity outcomes (no injury, minor injury, and severe injury) were modeled using variables related to the crash, driver, vehicle, and environment. The study found significant year-to-year variation in model parameters, particularly for gender and nighttime driving, while factors such as alcohol use, seatbelt use, older drivers, and airbag deployment showed stable effects. Temporal stability was assessed through likelihood ratio tests and marginal effect comparisons, highlighting the importance of accounting for heterogeneity and temporal shifts in injury severity modeling. *Islam, Liu*^8^ investigated crash severity factors in farm equipment vehicle crashes on county and non-county roads using a TabNet model. Drawing on a dataset that included variables such as road geometry, lighting, and traffic conditions, the study employed Synthetic Minority Over-sampling Technique (SMOTE) to address class imbalance and applied SHAP-based interpretability to understand feature importance. The results showed that crashes on county roads were primarily influenced by speed limit, first harmful event, traffic control, and driver age—highlighting the role of rural road geometry and demographic risk. In contrast, non-county road crashes were more affected by lighting conditions, intersection features, and population group, reflecting the greater complexity of urban traffic environments. Across all settings, speed limit consistently emerged as a dominant risk factor. The study recommends targeted safety measures such as improved visibility, speed regulation, and public education efforts tailored to road type and environment.

Based on the previous studies, while existing research has provided valuable insights into the characteristics and contributing factors of FEV-involved crashes, these studies generally fall into two categories: (i) descriptive analyses documenting crash patterns, and (ii) statistical or machine-learning models estimating the probability of injury severity. Collectively, these works show that FEV crashes tend to be more severe than other rural crashes and are influenced by roadway conditions, vehicle configurations, human behavior, and environmental factors.

However, despite these contributions, a clear research gap remains. Previous studies rarely incorporate modeling techniques that can capture systematic and random heterogeneity, even though FEV crashes occur in diverse roadway and operational contexts where unobserved variation is likely to be substantial. Moreover, the interaction effects among contributing factors—such as the combined influence of road geometry and vehicle characteristics on injury severity—have not been examined in prior FEV-related models.

By summarizing the state of knowledge and explicitly identifying these gaps, the revised Literature Review more clearly motivates the objectives of the present study and positions the methodological contributions of the RPOL and MC-SVM models within the broader crash-severity literature. The summary of the literature is presented in Table 1.

**Table 1:** Summary of the literature on FEV crash injury severity.

Author(s) (year)	Study focus	Key findings	Location/data	Methodology
*Peek-Asa, Sprince *^28^	Characteristics of crashes with farm equipment that increase injury potential	Non-farm drivers are 5 × more likely to be injured; lack of restraint use, speed, darkness, and passing increase injury odds; ejection is critical for farm driver injury	Iowa, 1995–2004 DOT crash data	Multinomial/Ordered Logit
*Costello, Schulman *^29^	Risk factors for farm vehicle public road crashes using a case–control study	Non-English speaking and non-family drivers, younger drivers, prior injury history, and public road sharing increase crash risk	North Carolina, 1992–2003 case–control survey	Regression Analysis
*Myers, Cole *^30^	Injury severity in tractor overturns comparing ROPS-equipped and non-ROPS tractors	ROPS significantly reduced fatal and severe injury outcomes by stopping overturns at 90°. Non-ROPS tractors had higher rates of continuous roll and associated injuries	Kentucky, U.S., 2002 survey of 6,063 farm operators	Multinomial Logit
*Gkritza, Kinzenbaw *^31^	Empirical analysis of injury severities in farm vehicle crashes using MNL model	Rear-end and single-vehicle crashes, older vehicles, younger drivers, rural settings, and nighttime driving increase severe injury odds	Iowa, 2004–2006 DOT crash data	Multinomial/Ordered Logit
*Pinzke, Nilsson *^32^	Age-related patterns in farm tractor crashes on Swedish public roads	Older drivers overrepresented in single-vehicle crashes; younger drivers involved in speed-related incidents. Severity higher in collisions with vulnerable road users or large vehicles	Sweden, 2003–2010 police-reported data	Multinomial Logit
*Harland, Greenan *^7^	Comparison of FEV crash characteristics by rural–urban location and town proximity	Urban/peri-urban crashes often at intersections with non-farm vehicles; rural crashes more likely to involve rollovers on high-speed roads	Midwestern U.S. states, 2005–2010 crash data	Multinomial Logit
*Anarkooli, Hosseinpour *^33^	Injury severity in single-vehicle rollover crashes using REGOP model	Dark unlit conditions, rain, undulating terrain, improper overtaking, older vehicles, and SUVs/trucks increase severity. Urban settings and wider unpaved shoulders reduce severity	Malaysia, 2007–2012 federal road crash data	Random-Effects Generalized Ordered Probit (REGOP)
*Harland, Bedford *^34^	Alcohol impairment and odds of driver injury/fatality in on-road farm equipment crashes	Alcohol-impaired drivers were overrepresented in fatal/injury crashes. Odds of injury/fatality were 4.1 × higher with alcohol impairment, especially among non-farm drivers	Great Plains U.S. states, 2005–2010 crash data	hierarchical logistic regression model
*Scott, Hirabayashi *^18^	Characteristics of FEV-related motor vehicle crashes in rural New York	FEV crashes had 5 × higher fatality rate than non-ag crashes; occurred mainly on straight graded roads in daylight; higher rates of moderate/severe injuries and two-vehicle crashes	New York State, 2010–2012 DMV crash data	Logistic Regression
*Cai, Wei *^35^	Modeling injury severity of low-visibility rural single-vehicle crashes with spatial correlation	Fog, curves, and early morning crashes increased injury severity. Spatial model showed regional heterogeneity; calls for localized countermeasures	Kansas, U.S., low-visibility crash data	Spatial Random Parameters Model
*Kim, Kim *^36^	Incidence rates and injury risks for various FEV types using insurance data	Injury risk highest for power carts and tillers; injury rate increasing annually. Elderly drivers at higher risk. Highlights need for machine-specific safety regulation	South Korea, 2014–2020 agricultural insurance data	Logistic Regression
*Ren, Xu *^37^	Factors affecting injury severity in rollover crashes	Temporal stability tests revealed year-to-year instability in model specifications but no significant aggregate-to-component variation	California, 2013–2017 HSIS crash data	Random Parameters Logit/RPOL
*Islam, Liu *^8^	Factors influencing crash severity, with a particular focus on comparing incidents on county roads to those on non-county roads	Road alignment and traffic control are crucial on county roads, lighting and intersections on non-county roads. Speed limits are a key factor influencing crash severity for county and non-county roads	2017–2022 TxDOT’s Crash Records Information System	TabNet (Machine Learning)

## Methodology

This study aims to analyze the injury severity outcomes of FEV-involved crashes on interurban roads by using a Random Parameters Ordered Logit (RPOL) model and a Multi-Class Support Vector Machine (MC-SVM) model. This approach captures both observed and unobserved heterogeneity, allowing for a more comprehensive assessment of how injury severity varies across collision configurations and roadway types.

The injury severity is modeled as an ordinal variable with three discrete levels: Property Damage Only (PDO = 1), Injury (2), and Fatal (3). Property Damage Only (PDO): A crash in which no road user sustains visible or reported physical injury; losses are limited to material or vehicle damage. Injury Crash: A crash resulting in at least one non-fatal injury to a road user, including minor, moderate, or severe injuries that require medical attention but do not lead to immediate or subsequent death. Fatal Crash: A crash in which at least one person dies within 30 days of the crash as a direct result of injuries sustained. The inclusion of both statistical and machine learning techniques allows for a comprehensive understanding of the contributing heterogeneous factors and examines the accuracy of different models in the prediction of injury severity for FEV-involved crashes.

### Random parameters ordered logit (RPOL) model

Traditional statistical models, like ordered probit or logit,assume fixed parameter effects, which may oversimplify the intricate and varied determinants of crash outcomes^38–40^.To capture the effects of both observed and unobserved heterogeneity, the Random Parameters Ordered Logit (RPOL) model is employed. For example, *Yu and Long*^41^ integrated environmental and vehicular condition data into Random Parameters Ordered Logit (RPOL) models, illustrating the benefits of flexible modeling frameworks to better represent real-world variability. Furthermore, the RPOL model allows for individual-level variation, improving interpretability and inference^42–44^. This model extends the traditional ordered logit framework by allowing parameter variation across observations, accounting for random heterogeneity and improving model flexibility and explanatory power^45–47^. The observed term is typically a function of explanatory variables, enabling the identification of heterogeneous impacts on crash severity. The random parameters are modeled to account for the variation across observations.

In the RPOL model, the latent dependent variable (i.e., crash severity) is expressed as a linear function of explanatory variables, along with a random error term. Equation (1) below represents this relationship:

1 $$Y_{i}^{*} = \alpha X_{i} + \beta^{\prime}X_{i} + \vartheta_{i}$$

where:

${\mathrm{Y}}_{{\mathrm{i}}}^{*}$ is the latent severity of a crash in observation *i*,

${\mathrm{X}}_{{\mathrm{i}}}$ is a (*L* × *1*) vector of observed explanatory variables for observation *i*,

${\upalpha }$ is a (1 × L) vector of non-random coefficients for explanatory variables,

$\beta^{\prime}$ is a (*1* × *L*) vector of random coefficients for explanatory variables,

$\vartheta_{i}$ is the error term for observation *i*, assumed to follow an independent and identically distributed (IID) Gumbel distribution.

The random parameters consist of two components:

1. An observed term containing a constant *β* and a relation with the explanatory variables $\Gamma X_{i}$ (Eq. 2),
2. An unobserved term, assumed to follow a random distribution (e.g., Normal distribution).

Equation (2) illustrates the structure of the random parameters:

2 $$\beta^{\prime} = \beta + \Gamma X_{i} + \varepsilon$$

where:

${\rm B}$ is a (*L* × *1*) vector of constant coefficients, L is the number of random parameters,

$\Gamma$ is a (*L* × *L*) matrix of mean coefficient for explanatory variables,

$\varepsilon$ is a (*L* × *1*) vector of error terms, assumed to be normally distributed with zero mean.

Assuming that $\varepsilon$ is normally distributed with zero vector of constants (*L* × *1*), diagonal matrix of covariance ${\uppsi }$ (*L* × *L*), Probability Distribution Function (PDF) related with random parameters can be formulated as Eq. (3).

3 $$f\left( {\beta^{\prime}\left| {X_{i} } \right.;\beta ,\Gamma ,\psi } \right) = (2\pi )^{{ - \frac{1}{R}}} \left| \psi \right|^{{ - \frac{1}{2}}} \exp \left( { - \frac{1}{2}\left( {\beta^{\prime} - \beta + \Gamma X_{i} } \right)^{T} \psi^{ - 1} (\beta^{\prime} - \beta + \Gamma X_{i} )} \right)$$

The latent variable ${\mathrm{Y}}_{{\mathrm{i}}}^{*}$ can be converted to an ordinal variable ${\mathrm{Y}}_{{\mathrm{i}}}$ with respect to τ intervals. In the other words, ${\mathrm{Y}}_{{\mathrm{i}}}^{*}$ represents the latent variable for crash severity, which is influenced by observed and random factors. The variable ${\text{ Y}}_{{\mathrm{i}}}$ is the discretized (predicted) ordered variable for crash severity, which is derived by applying estimated thresholds to ${\mathrm{Y}}_{{\mathrm{i}}}^{*}$*.* The ${\text{ Y}}_{{\mathrm{i}}}$ can be formulated as Eq. (4).

4 $$Y_{i} = \left\{ {\begin{array}{*{20}c} {1,\quad if\,\alpha X_{i} + \beta^{\prime}X_{i} + \varepsilon_{i} \le \tau_{1} } \\ {2,\quad if\,\tau_{1} \le \alpha X_{i} + \beta^{\prime}X_{i} + \varepsilon_{i} \le \tau_{2} } \\ {3,\quad if\,\tau_{2} \le \alpha X_{i} + \beta^{\prime}X_{i} + \varepsilon_{i} } \\ \end{array} } \right.\quad \forall \tau_{j}> \tau_{j - 1}$$

where ${\text{ j }} \in { }\left\{ {1, 2, 3} \right\}$ denotes the predicted level of crash severity. *Y*_*i*_, with “*1*” denoting PDO crash “2” denoting injury crash, and “3” denoting fatal crash.

5 $$Y_{i} = \left\{ \begin{gathered} 1,PDO\,crash \hfill \\ 2,Injury\,crash \hfill \\ 3,Fatal\,crash \hfill \\ \end{gathered} \right.$$

The probability of each discrete number $j$ can be written as Eq. (6) to (10):

6 $$P\left( {Y_{i} = 1|X_{i} } \right) = P\left( {Y_{i}^{*} < \tau_{1} |X_{i} } \right) = \Phi \left( {\tau_{1} } \right)$$

7 $$P\left( {Y_{i} = 2|X_{i} } \right) = P\left( {\tau_{1} \le Y_{i}^{*} < \tau_{2} |X_{i} } \right) = \Phi \left( {\tau_{2} } \right) - \Phi \left( {\tau_{1} } \right)$$

8 $$P\left( {Y_{i} = 3|X_{i} } \right) = P\left( {\tau_{2} \le Y_{i}^{*} |X_{i} } \right) = 1 - \Phi \left( {\tau_{2} } \right)$$

where $${\Phi }$$($${.)}$$ is the Cumulative Distribution Function (CDF) assumed to have a Gumbel distribution.

The likelihood function for the observation *i*^*th*^ is presented in Eq. (9).

9 $$P\left( {Y_{i} \left| {X_{i} ;\beta^{\prime } \vartheta_{i} } \right.} \right) = \mathop \Pi \limits_{j = 1}^{3} P\left( {Y_{i} = j|X_{i} } \right)^{{m_{ij} }}$$

where, ${\mathrm{m}}_{{{\mathrm{ij}}}}$ is equal to *1* if ${\mathrm{Y}}_{{\mathrm{i}}}$ = *j*, otherwise, equal to *0*. Subsequently, the joint PDF for the discrete level of crash severity and randomness of parameters can be formulated as Eq. (10).

10 $$f_{{i \times \beta^{\prime } }} \left( {Y_{i} ,\beta^{\prime } \left| {X_{i} ;\beta \Gamma \psi \vartheta_{i} } \right.} \right) = \int_{{\beta^{\prime } }} {\left( {P\left( {Y_{i} |X_{i} ;\beta^{\prime } ,\vartheta_{i} } \right)} \right)f\left( {\beta^{\prime } |X_{i} ;\beta \Gamma \psi } \right)d\beta^{\prime } }$$

Finally, the log-likelihood function, which accounts for the joint probability of crash severity and parameter heterogeneity, is expressed as:

11 $$LL = \sum\limits_{i = 1}^{I} {Log\left( {f_{{i \times \beta^{\prime } }} \left( {Y_{i} \beta^{\prime } |X_{i} ;\beta \psi \vartheta_{i} } \right)} \right)}$$

The parameters are estimated by maximizing the log-likelihood function (Eq. 13) across the sample^48^.

### Multi-class support vector machine (MC-SVM) model

In addition to the RPOL model, this study used a Multi-Class Support Vector Machine (MC-SVM) to predict injury severity outcomes of FEV-involved crashes that were categorized into three classes: PDO (i.e., level 1), injury (i.e., 2), and fatal (i.e., 3). To handle the multi-class classification task, the Crammer-Singer method was adopted, which extends the binary outcome formulation to the multi-class case^49^. The objective function for the MC-SVM is defined as Eq. (12):

12 $$\frac{{1}}{{2}}\mathop \sum \limits_{{\text{j = 1}}}^{{3}} {\mathrm{w}}_{{\mathrm{j}}}^{{2}} {\text{ + C}}\mathop \sum \limits_{{\text{i = 1}}}^{{\mathrm{N}}} {\upxi }_{{\mathrm{i}}}$$

where:

${\mathrm{w}}_{{\mathrm{j}}}$ weight vector for the *j*-th class (injury severity level),

*C*regularization parameter,

${\upxi }_{{\mathrm{i}}} { } \ge { 0}$ slack variable penalizing margin violations for the *i*-th observation.

Given training data $\left\{ {x_{i} , y_{i} } \right\}\,\forall \,i = 1,2, \ldots ,N$ where, ${\mathrm{y}}_{{\mathrm{i}}} { } \in { }\left\{ {1, 2, 3} \right\}$, the constraint condition ensures that the score of the true class ${\mathrm{y}}_{{\mathrm{i}}}$ is larger than that of all other classes ${\mathrm{j}} \ne {\mathrm{y}}_{{\mathrm{i}}}$, and expressed as Eq. (13):

13 $$w_{{y_{i} }} \cdot x_{i} + b_{{y_{i} }} \ge w_{j} \cdot x_{i} + b_{j} + 1 - \xi_{i} \forall j \ne y_{i}$$

where:

${\mathrm{b}}_{{\mathrm{j}}}$ bias term for the *j*-th injury severity class,

${\mathrm{x}}_{{\mathrm{i}}}$ is the vector of observed variables for observation i that affect the injury severity,

${\mathrm{y}}_{{\mathrm{i}}}$ is the class of accident injury severity, for PDO accidents ${\text{ y}}_{{\mathrm{i}}}$ = *1*, injury accidents ${\mathrm{y}}_{{\mathrm{i}}}$ = *2*, and for fatal accidents ${\mathrm{y}}_{{\mathrm{i}}}$ = *3*.

This constraint ensures the score of the true class $y_{i}$ exceeds the score of every other class *j* by at least $1 - \xi_{i}$. For a new accident input *x*, predicting the class j with the highest score can be formulated as Eq. (14).

14 $${\text{y = arg max ( w}}_{{\mathrm{j}}} { } \cdot {\text{ x}}_{{\mathrm{i}}} {\text{ + b}}_{{\mathrm{j}}} {) }\forall {\text{ j }} \in { }\left\{ {1, 2, 3} \right\}{ }$$

This model enables robust classification by optimizing the separation between all severity levels and accounting for the inter-class margin constraints. The incorporation of MC-SVM alongside RPOL offers a comparative perspective between a probabilistic parametric approach and a data-driven machine learning classifier in modeling injury severity.

## Case study

This study investigates FEV-involved crashes in Iran, a country encompassing approximately 1.6 million square kilometers with a population of 89 million. Iran has an extensive transportation infrastructure, comprising 88,873 km of interurban roads and 132,317 km of rural roads. Despite this infrastructure, the country experiences a persistently high rate of road traffic fatalities. Between March 2023 and March 2024 alone, 20,045 people were killed and 391,069 injured in traffic incidents occurring on interurban and rural roads^50^.

Based on the 2024 crash data in Iran, out of 284 FEV-involved crashes, 57 were fatal (20%) and 164 resulted in injuries (58%). For comparison, considering all vehicle types in interurban roads in Iran, out of 82,503 total crashes, 6,417 were fatal (7.77%) and 45,565 resulted in injuries (55%). These statistics demonstrate that FEV-involved crashes have a substantially higher fatality rate compared to the overall crash population. On the other hand, FEV-involved crashes are particularly severe in Iran. Approximately 20% of these incidents result in fatalities—an alarmingly high figure when compared to global benchmarks. For instance, international studies report fatality rates of only about 2% in FEV-involved crashes^10,28,51^. This stark contrast underscores the importance of investigating contextual risk factors and road safety conditions specific to Iran.

To that end, this research utilizes a comprehensive dataset of FEV-involved crashes in Iran between 2019 and 2024. The dataset includes 768 recorded FEV-involved crashes, spanning the inter-urban road network. Supplementary data from Iran’s traffic surveillance systems, which document driver violations and enforcement activities, are also incorporated to enhance the explanatory power of the analysis^50^.

**Fig. 1:**
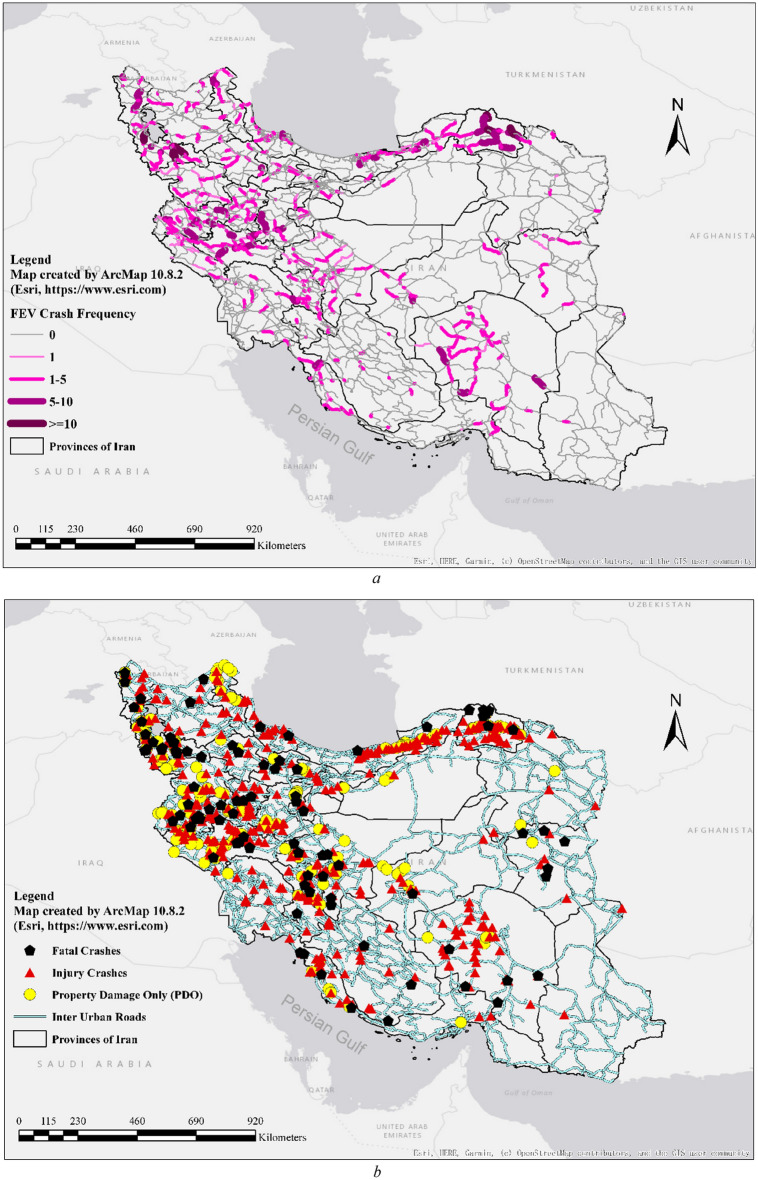
Locations of FEV-involved crashes in Iran (2019 to 2024), Map created by the authors using ArcMap 10.8.2 (Esri, https://www.esri.com).

Figure 1 presents the spatial distribution of FEV-involved crashes across Iran’s provinces, differentiated by road segment and topographic characteristics. The figure reveals a notable concentration of crashes in western regions of the country. This spatial clustering likely reflects higher population densities, more intensive FEV activity, and greater transport of farm products in those areas, which together elevate the exposure risk for such crashes.**.**

### Data description

Table 2 presents the descriptive statistics of 768 recorded FEV-involved crashes in Iran between 2019 and 2024. Of these, 22.01% were categorized as property damage only (PDO), 58.33% resulted in injuries, and 19.66% were fatal crashes. The relatively high proportion of fatal outcomes underscores the elevated risk associated with FEV use on public roadways, particularly in a developing country context.

**Table 2:** FEV-involved crash data descriptive statistics (2019 to 2024).

	Variable	Crashes N (% of total)	PDO Crashes N (% of total)	Injury Crashes N (% of total)	Fatal Crashes N (% of total)
Hour	0:00–6:00	52 (6.77%)	9 (5.33%)	25 (5.58%)	18 (11.92%)
	6:00–12:00	210 (27.34%)	59 (34.91%)	113 (25.22%)	38 (25.17%)
	12:00–18:00	249 (32.42%)	65 (38.46%)	137 (30.58%)	47 (31.13%)
	18:00–24:00	257 (33.46%)	36 (21.3%)	173 (38.62%)	48 (31.79%)
Weather	Sunny	756 (98.44%)	166 (98.22%)	440 (98.21%)	150 (99.34%)
	Rainy	2 (0.26%)	1 (0.59%)	1 (0.22%)	0 (0.0%)
	Snowy	2 (0.26%)	1 (0.59%)	1 (0.22%)	0 (0.0%)
	Cloudy	8 (1.04%)	1 (0.59%)	6 (1.34%)	1 (0.66%)
Accident Type	Overturn and fall	108 (14.06%)	12 (7.1%)	48 (10.71%)	48 (31.79%)
	One vehicle	483 (62.89%)	136 (80.47%)	283 (63.17%)	64 (42.38%)
	With fixed object	12 (1.56%)	7 (4.14%)	3 (0.67%)	2 (1.32%)
	Multiple vehicles	89 (11.59%)	8 (4.74%)	63 (14.06%)	18 (11.92%)
	With pedestrian	23 (2.99%)	1 (0.59%)	14 (3.12%)	8 (5.3%)
	Other	53 (6.90%)	5 (2.96%)	37 (8.26%)	11 (7.28%)
Collision Type	Front to back	260 (33.85%)	64 (37.87%)	175 (39.06%)	21 (13.91%)
	Sliding out of road	56 (7.29%)	7 (4.14%)	27 (6.03%)	22 (14.57%)
	Front to front	174 (22.66%)	50 (29.59%)	80 (17.86%)	44 (29.14%)
	Front to side	177 (23.05%)	35 (20.71%)	115 (25.67%)	27 (17.88%)
	Upside down	65 (8.46%)	7 (4.14%)	28 (6.25%)	30 (19.87%)
	Side to side	22 (2.86%)	1 (0.59%)	14 (3.12%)	7 (4.64%)
	Other	14 (1.82%)	5 (2.96%)	9 (2.01%)	0 (0.0%)
Accident Cause	Not paying attention to the front	347 (45.18%)	64 (37.87%)	222 (49.55%)	61 (40.4%)
	Inability to control the vehicle	68 (8.85%)	16 (9.47%)	24 (5.36%)	28 (18.54%)
	Exceeding the safe speed	31 (4.04%)	3 (1.78%)	20 (4.46%)	8 (5.3%)
	Left deviation	59 (7.68%)	13 (7.69%)	36 (8.04%)	10 (6.62%)
	Not respecting the priority	91 (11.85%)	17 (10.06%)	58 (12.95%)	16 (10.6%)
	Not respecting the distance	28 (3.65%)	11 (6.51%)	15 (3.35%)	2 (1.32%)
	Sudden lane change	18 (2.34%)	6 (3.55%)	10 (2.23%)	2 (1.32%)
	Lack of alertness	3 (0.39%)	1 (0.59%)	1 (0.22%)	1 (0.66%)
	Vehicle defect	23 (2.99%)	1 (0.59%)	15 (3.35%)	7 (4.64%)
	Reverse gear movement	18 (2.34%)	13 (7.69%)	4 (0.89%)	1 (0.66%)
	Moving in the opposite direction	10 (1.3%)	2 (1.18%)	8 (1.79%)	0 (0.0%)
	Unauthorized overtaking	5 (0.65%)	1 (0.59%)	2 (0.45%)	2 (1.32%)
	Turning around in a prohibited area	5 (0.65%)	1 (0.59%)	3 (0.67%)	1 (0.66%)
	Other	62 (8.07%)	20 (11.83%)	30 (6.7%)	12 (7.95%)
Second involved vehicle	Truck	300 (77.12%)	85 (28.33%)	159 (53%)	56 (18.67%)
	Bus	11 (2.83%)	3 (27.27%)	7 (63.64%)	1 (9.09%)
	Motorcycle	78 (20.05%)	4 (36.36%)	57 (73.08%)	17 (21.79%)
Lighting	Daylight	429 (55.86%)	116 (68.64%)	228 (50.89%)	85 (56.29%)
	Dark	145 (18.88%)	26 (15.38%)	87 (19.42%)	32 (21.19%)
	Dusk/dawn	194 (25.26%)	27 (15.98%)	133 (29.69%)	34 (22.52%)
Day	Weekend	221 (28.78%)	50 (29.59%)	127 (28.35%)	44 (29.14%)
	Not_Weekend	547 (71.22%)	119 (70.41%)	321 (71.65%)	107 (70.86%)
	Holiday	163 (21.22%)	31 (18.34%)	103 (22.99%)	29 (19.21%)
Pavement	Acceptable	489 (63.67%)	108 (63.91%)	293 (65.4%)	88 (58.28%)
	Lowfailure	245 (31.9%)	57 (33.73%)	134 (29.91%)	54 (35.76%)
	Moderatefailure	28 (3.65%)	4 (2.37%)	17 (3.79%)	7 (4.64%)
	Severefailure	6 (0.78%)	0 (0.0%)	4 (0.89%)	2 (1.32%)
Total PDO Crashes	169 (22.01%)				
Total Injury Crashes	448 (58.33%)				
Total Fatal Crashes	151 (19.66%)				
Total Crashes	768 (100%)				

It should be noted that, most of crashes occurred frequently during the afternoon (12:00–18:00, 32.42%) and evening (18:00–24:00, 33.46%). Notably, fatal crashes were more concentrated in the evening (31.79%) and late afternoon (31.13%), suggesting that reduced visibility, traffic congestion, and operator fatigue during these periods may contribute to increased crash severity. Weather conditions were overwhelmingly favorable in most cases, with 98.44% of crashes occurring under sunny or clear skies, a statistic consistent with Iran’s predominantly dry and arid climate^52,53^. Rainy and snowy weather conditions accounted for only 0.26% each, indicating that environmental factors such as precipitation played a minimal role in crash occurrence or severity. When disaggregated by accident type, the most prevalent were single-vehicle crashes, which accounted for 62.89% of all cases and 42.38% of fatal crashes. “Overturn and fall” incidents comprised only 14.06% of all crashes but represented a disproportionate 31.79% of fatalities, suggesting these events are particularly severe. Multi-vehicle crashes accounted for 11.59% of total incidents and 11.92% of fatalities, highlighting the dangers posed by interactions between FEV and non-FEVs.

Collision type also played a significant role in injury severity. Front-to-back collisions were the most common (33.85%) but were responsible for only 13.91% of fatal crashes. In contrast, front-to-front (head-on) collisions and sliding off the road were both associated with higher fatality rates—29.14% and 14.57%, respectively. Upside-down (rollover) collisions, while less frequent (8.46%), accounted for 19.87% of fatalities, reinforcing the high lethality of vehicle instability or control loss events. The analysis of crash causation revealed that failure to pay attention to the road ahead was the leading cause, responsible for 45.18% of crashes and 40.4% of fatalities. Inability to control the vehicle contributed to 8.85% of total incidents but was associated with 18.54% of fatal crashes, highlighting it as a high-risk behavior. Other notable causes included failure to yield the right of way (11.85%) and exceeding safe speed limits (4.04%). In terms of the second most common vehicle that collided with FEVs, the majority of accidents (77.12%) were caused by collisions with heavy vehicles and trucks. The fatality rate in this group was 18.67%. Also, collisions between FEVs and motorcycles on rural roads accounted for about 20% of the accidents, with a fatality rate of 21.79%. Lighting conditions were also a critical factor. While most crashes occurred during daylight hours (55.86%), dusk/dawn and nighttime conditions were disproportionately associated with fatalities, comprising 43.71% of all fatal outcomes. This pattern suggests that limited visibility during transitional lighting periods significantly heightens crash severity risk.

The pavement condition variable used in this study was obtained from Iran’s national Pavement Management System (PMS), which is maintained by the Ministry of Roads and Urban Development. PMS assessments combine two standard engineering indicators—the Pavement Condition Index (PCI) and the International Roughness Index (IRI)—to evaluate pavement integrity and assign each road segment to a maintenance-oriented condition category. Based on the joint interpretation of PCI and IRI values by pavement experts, each crash location in the accident database was classified into one of four pavement-condition levels: Acceptable, Low Failure, Moderate Failure, or Severe Failure. These categories reflect the relative degree of pavement deterioration and the corresponding level of maintenance or rehabilitation required. The specific PCI and IRI thresholds associated with each category are presented in Table 3, and the same classification scheme was used to define the pavement-quality variable in the modeling dataset.

**Table 3:** Thresholds considered to determine variable levels of pavement quality.

**PCI**	*900–100*	*90–60*	*60–40*	*PCI* < *40*
IRI	*0–3*	*0–3*	*3–4*	*IRI* > *4*
Pavement Condition	*Acceptable*	*Low failure*	*Moderate failure*	*Severe failure*

According to PMS statistics reported in 2024, approximately 24% of the national road network fell into the Acceptable category, 35% into Low Failure, 26% into Moderate Failure, and 15% into Severe Failure. Pavement-condition analysis shows that although most FEV crashes occurred on Acceptable roads, the severity rate increased noticeably as pavement quality declined. Crashes on Low Failure, Moderate Failure, and Severe Failure segments—despite lower exposure—were associated with a disproportionately higher share of fatal outcomes. This pattern highlights the critical role of timely pavement maintenance in reducing crash severity.

In conclusion, the descriptive analysis identifies a consistent pattern of high injury and fatality risk associated with rollover-type crashes, loss of control, poor driver attention, and nighttime or evening driving. These factors informed the input selection for the subsequent RPOL and MC-SVM modeling approaches, which aim to quantify and predict injury severity based on crash characteristics and contextual variables.

### Data bank and feature selection

To evaluate the predictive performance of the developed models, the full dataset of 768 FEV-involved crash records was randomly partitioned into a training set (80%, or 615 observations) and a test set (20%, or 153 observations). This approach allows both the RPOL model and the MC-SVM model to be trained on a robust dataset while assessing their out-of-sample predictive validity using the test set. The use of this holdout validation method aligns with best practices in crash severity modeling and machine learning applications^54,55^.

Prior to modeling, a correlation analysis was conducted to evaluate the relationship between each candidate explanatory variable and the outcome variable (i.e., crash severity). Variables exhibiting multicollinearity or lacking significant association with injury severity were excluded from the final modeling specifications. This step ensured greater model interpretability and minimized estimation bias. The selected features encompassed a diverse range of crash-related characteristics, collision configurations, road geometry, environmental conditions, and behavioral attributes, which were subsequently used to train and validate both the RPOL and MC-SVM models. For the implementation of RPOL modeling, we used the NumPy, SciPy, Pandas, and Biogeme libraries in Python. For MC-SVM, we used the Pandas, NumPy, and Scikit-learn libraries.

## Results

This section presents the modeling results for the Random Parameters Ordered Logit (RPOL) and MC-SVM models, both developed to evaluate injury severity outcomes in FEV-involved crashes.

### RPOL model specification

The RPOL model was constructed according to the formulation introduced in Section "Methodology". The latent crash severity variable ${\mathrm{Y}}_{{\mathrm{n}}}^{*}$ was modeled as a linear function of multiple explanatory variables, some of which were treated as random parameters to capture unobserved heterogeneity. Equation (15) defines the model structure:

15 $$\begin{aligned} Y_{n}^{*} & = \beta_{0} + \beta_{1} Unsafe\_Dis\tan ce\_cause_{n} + \beta_{2} Vehicle\_Defect\_Cause_{n} \\ & + \beta_{3} {\mathrm{Re}} ver\sin g\_Cause_{n} + \beta_{4} Overturn\_Occurred_{n} + \beta_{5} Collision\_with\_Fixed\_Object_{n} \\ & + \beta_{6} Accident\_0000\_to\_0600_{n} + \beta_{7} Truck\_Involved_{n} + \beta_{8} Motorcycle\_Involved_{n} \\ & + \beta_{9} {\mathrm{Sin}} gle\_Lane\_Road_{n} + \beta_{10}{\prime} Front\_to\_Back\_Collision_{n} + \vartheta_{n} \\ \end{aligned}$$

where ${\mathrm{Y}}_{{\mathrm{n}}}^{*}$ is the latent variable of crash severity, ${\upbeta }_{{0}}$ to ${\upbeta }_{{9}}$ are non-random parameters of the model and ${\upbeta }_{{{10}}}{\prime}$ is the random parameters of the RPOL model and $\vartheta_{{\mathrm{n}}}$ is the random part of the latent variable of crash severity, which is assumed to have a Gamble distribution function with zero mean. The definition of other parameters is presented in Table 2. The random parameters of the RPOL model is defined as shown in Eqs. (16) show. It must be noted that in this study, these equations are chosen based on reaching the best Akaike Information Criterion (AIC) and Bayesian Information Criterion (BIC) of the RPOL model.

16 $$\beta^{\prime}_{10} = a_{0} + a_{1} FEV\_at\_Fault_{n} + a_{2} Straight\_Road_{n} + \varepsilon_{n}$$

where ${\mathrm{a}}_{i} { }\left( {{\text{i }} \in \left\{ {0, 1, 2} \right\}} \right)$ is the parameter of the systematic heterogeneity of the random parameters and ${\upvarepsilon }_{{\mathrm{n}}}$ is a random term of random parameters that are assumed to be Identically and Independently Distributed (IID) based on the Normal distribution function with zero mean. Other variables are defined in Table 2.

### RPOL and MC-SVM results

Using the training subset of 615 observations, the RPOL model was calibrated based on model selection criteria such as the AIC and BIC. Table 4 presents the final estimation results, including both fixed and random parameters.

**Table 4:** RPOL model for the severity of the FEV-involved crashes5.3. Model Evaluation and Comparative Analysis.

Variable	Parameter	Coef	z	P > z
The constant of the injury severity function	*Constant*	0.07	*0.669*	*0.50*
Failure to maintain safe longitudinal/lateral distance as accident cause (yes = 1, and otherwise = 0)	*Unsafe_Distance_cause*	−1.35	*−3.1*	*0.00*
Vehicle technical defect as accident cause (yes = 1, and otherwise = 0)	*Vehicle_Defect_Cause*	1.1	*2.08*	*0.04*
Reversing movement as accident cause (yes = 1, and otherwise = 0)	*Reversing_Cause*	−3.16	*−4.35*	*0.00*
Overturn/rollover occurred in the accident (yes = 1, and otherwise = 0)	*Overturn_Occurred*	1.25	*4.18*	*0.00*
Vehicle collided with a fixed object (yes = 1, and otherwise = 0)	*Collision_with_Fixed_Object*	−1.87	*−2.08*	*0.04*
Accident occurred between 00:00–06:00 h (yes = 1, and otherwise = 0)	*Accident_0000_to_0600*	1.12	*2.89*	*0.00*
Truck was involved in the accident (yes = 1, and otherwise = 0)	*Truck_Involved*	−2.2	*−2.68*	*0.01*
Motorcycle was involved in the accident (yes = 1, and otherwise = 0)	*Motorcycle_Involved*	1.13	*4.04*	*0.00*
Accident occurred on a single-lane road (yes = 1, and otherwise = 0)	*Single_Lane_Road*	0.391	*1.93*	*0.05*
Collision type was front-to-back (yes = 1, and otherwise = 0)	*Front_to_Back_Collision*	−0.99	*−2.74*	*0.01*
Heterogeneity in the mean of parameters				
FEV/tractor was the primary at-fault party (yes = 1, and otherwise = 0)	*Front_to_Back_Collision: FEV _at_Fault*	*−0.74*	*−2.25*	*0.02*
Accident occurred on a straight road segment with no curves, intersections, or vertical curves (yes = 1, and otherwise = 0)	*Front_to_Back_Collision: Straight_Road*	*0.913*	*2.55*	*0.01*
*tau_1* = *−1.07*				
*tau_2* = *3.18*				
The number of obs. = 615 (Train set size)				
Init log likelihood = −799.48				
Final log likelihood = −545.12				
Rho-square = 0.318				
Rho-square-bar = 0.299				
Akaike Information Criterion (AIC) = 1120.24				
Bayesian Information Criterion (BIC) = 1186.57				

Table 4 presents the estimation results of the final RPOL model. This model incorporates both fixed and random parameters, as specified in Section "RPOL Model Specification", and accounts for heterogeneity in crash severity outcomes based on crash characteristics, vehicle attributes, and environmental conditions. The inclusion of random parameters allows the model to flexibly capture unobserved heterogeneity, providing a more nuanced understanding of the factors influencing the severity of FEV-involved crashes in Iran.

Key findings from the RPOL model indicate that rollover crashes, involvement of motorcycles, nighttime crashes (00:00–06:00), and vehicle defects significantly increase the likelihood of severe injuries or fatalities. In contrast, rear-end collisions and reversing-related crashes were associated with lower severity levels. The inclusion of interaction effects (e.g., FEV at fault × front-to-back collisions) enhanced the model’s capacity to capture situational variability in severity outcomes. The model yielded an AIC of 1120.24 and a BIC of 1186.57, with a pseudo R-squared of 0.318, indicating an acceptable level of explanatory power.

**Fig. 2:**
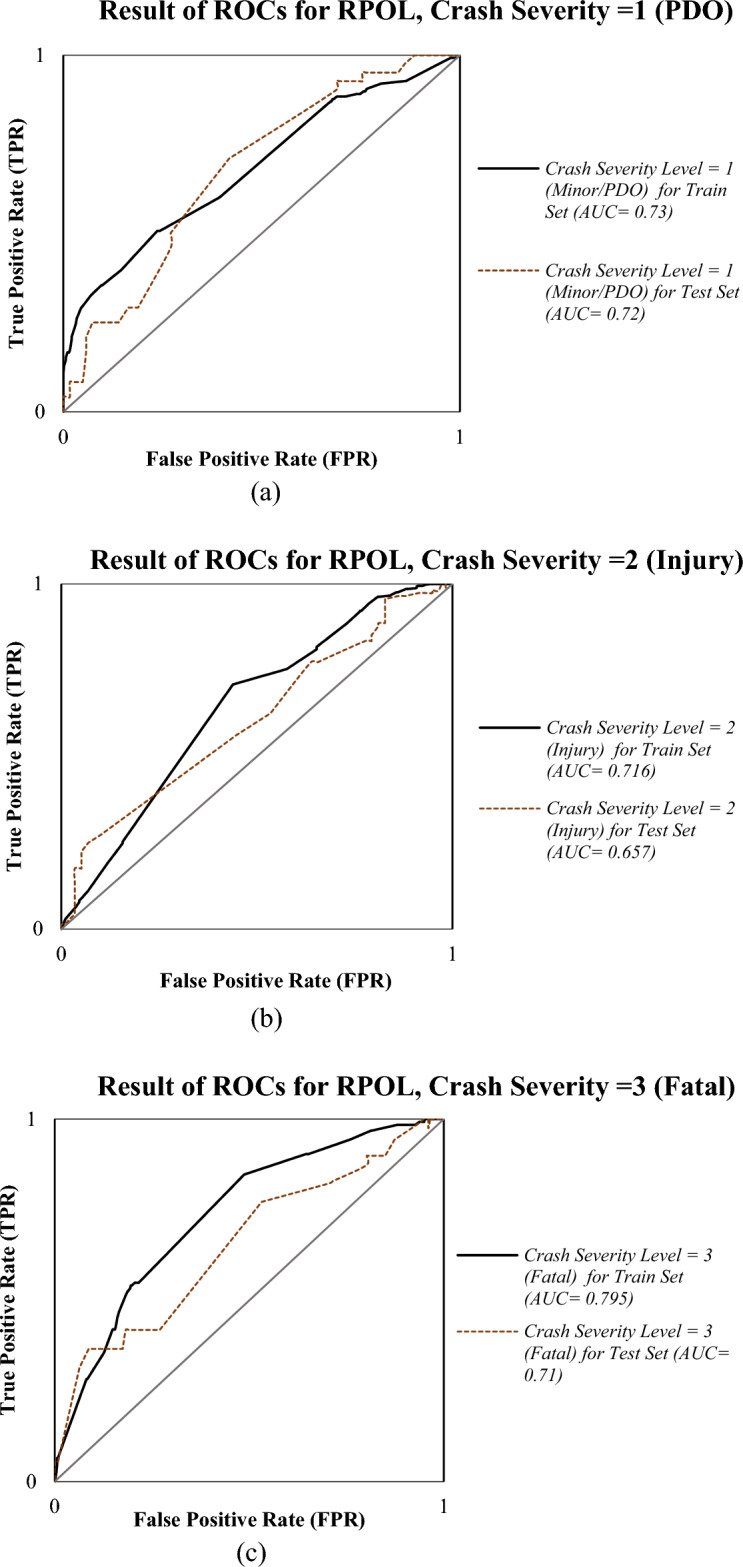
The ROCs for RPOL model.

To evaluate its classification performance, ROC curves were generated using predicted probabilities across the three severity classes. Figure 2 illustrates the ROC curves for both training and test datasets of RPOL model. The model displayed s high AUC values and a potential ability to distinguish between PDO, injury, and fatal crash categories.

**Fig. 3:**
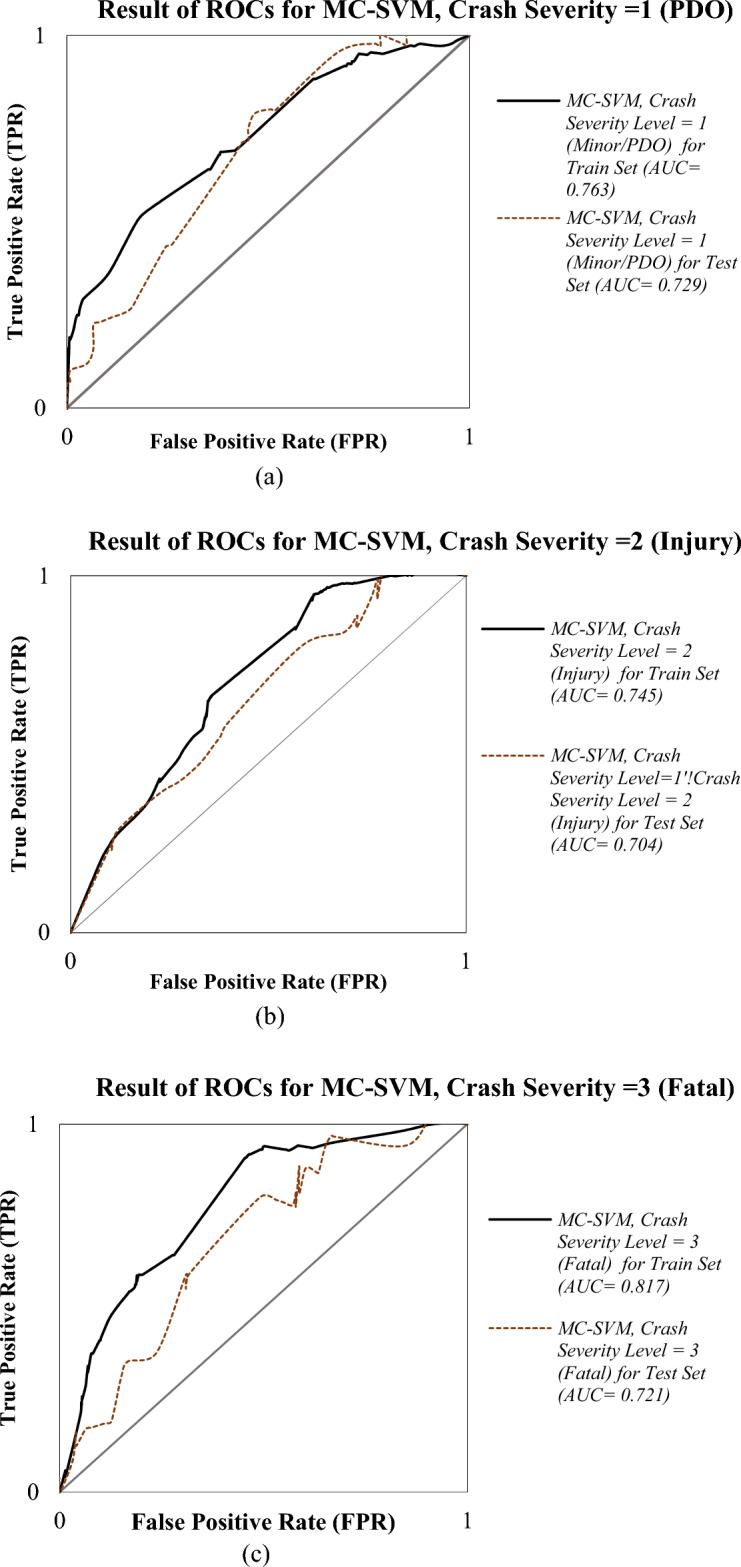
The ROCs for MC-SVM.

In parallel, the MC-SVM model was trained using the same feature set and training dataset. After parameter tuning and cross-validation, the final MC-SVM classifier achieved robust classification accuracy. The model demonstrated high predictive precision, particularly in identifying fatal crashes, which are often underrepresented in imbalanced datasets. Figure 3 demonstrates the ROC curves for the MC-SVM model’s training and test datasets. Comparative analysis between RPOL and MC-SVM models showed that while RPOL offers interpretability and parametric insight, the MC-SVM model provided enhanced prediction performance, especially in correctly classifying injury and fatal cases. This complementary performance underscores the value of using both statistical and machine learning approaches in traffic crash severity analysis.

Based on the ROC curve results illustrated in Figs. 2 and 3, both the RPOL and MC-SVM models exhibit comparable accuracy in predicting PDO and Injury crash severity outcomes across the training and testing datasets. However, the MC-SVM model demonstrates superior generalization capability, achieving consistently higher AUC values across all injury severity levels.

According the results, MC-SVM achieved test AUCs of 0.729 for PDO, 0.740 for Injury, and 0.721 for Fatal crashes, outperforming RPOL’s corresponding values of 0.720, 0.657, and 0.710, respectively. The most pronounced improvement was observed in the Injury category, where MC-SVM exceeded RPOL by 12.6% (ΔAUC = 0.083). It seems MC-SVM’s kernel-based approach (using a radial basis function kernel in our implementation) allows it to map input features into a higher-dimensional space, enabling the model to detect non-linear patterns that linear or parametric models like RPOL might overlook, such as threshold effects in speed differentials or multiplicative interactions between time of day and road geometry, and probably this issue lead to more predictive power of the MC-SVM. Although both models showed signs of overfitting, the degradation in performance from training to testing was less severe in MC-SVM. The average AUC drop for MC-SVM was 0.045, compared to 0.051 for RPOL. For PDO outcomes, MC-SVM displayed slightly better performance during both training (AUC = 0.763) and testing (AUC = 0.729) than RPOL (training = 0.730; testing = 0.720). The RPOL model maintained more consistent training and testing AUCs for PDO (ΔAUC = 0.010) than MC-SVM (ΔAUC = 0.034), though MC-SVM’s test accuracy remained superior.

For Injury outcomes, MC-SVM demonstrated exceptional stability, with nearly identical training (AUC = 0.745) and testing (AUC = 0.740) scores, indicating minimal overfitting (ΔAUC = 0.005). Conversely, RPOL’s Injury prediction performance suffered significant degradation from training (AUC = 0.716) to testing (AUC = 0.657), reflecting a 0.059 decrease. This further confirms MC-SVM’s advantage in modeling mid-range severity outcomes.

Fatal outcomes posed a generalization challenge for both models. RPOL experienced an AUC drop of 0.085 (training = 0.795 → testing = 0.710), while MC-SVM recorded a similar reduction of 0.096 (training = 0.817 → testing = 0.721). Despite this, MC-SVM maintained a slight edge in test performance (0.721 vs. 0.710), underscoring its marginally better robustness in classifying severe crashes.

Overall, the MC-SVM model exhibited significantly lower performance degradation across severity classes (mean ΔAUC = 0.045) compared to RPOL (mean ΔAUC = 0.051). The Injury category (Class 2) proved to be the most stable and accurately predicted class for MC-SVM, while both models struggled to generalize for Fatal outcomes (Class 3). Besides, RPOL’s vulnerability to overfitting was particularly acute in Classes 2 and 3, where test performance fell below acceptable thresholds (AUC < 0.700).

### Interpretation of RPOL estimation results

According to the results shown in Table 4, the *Unsafe_Distance_Cause* variable indicates that failure to maintain safe longitudinal/lateral distance reduces injury severity. This counterintuitive effect likely arises because such crashes (e.g., rear-end collisions) often occur at lower speed differentials in congested rural traffic. The farm vehicle’s rigid structure may absorb impact forces more effectively than in high-energy collisions, and seatbelt usage (though inconsistent) may mitigate occupant ejection. However, this does not imply safety—it reflects relative severity compared to catastrophic crash types like rollovers.

The results show that *Vehicle_Defect_Cause* signifies that technical defects (e.g., brake/steering failure) increase severity. Farm vehicles with mechanical failures often lose control abruptly on rural roads, leading to high-speed collisions or rollovers. Defects compromise crash-avoidance capabilities and structural integrity, exacerbating impacts—especially in aging vehicle fleets common in developing countries. Prior studies have also explored the link between vehicle defects and crash severity^56,57^. Furthermore, according to Table 4, the negative sign of *Reversing_Cause* shows that reversing-related crashes reduce severity. These incidents typically involve low-speed impacts (< 15 km/h) during farm activities (e.g., backing near fields). The minimal kinetic energy transfer rarely causes structural deformation or occupant trauma, though pedestrians/cyclists remain at risk if struck.

As illustrated in Table 4, the positive sign of *Overturn_Occurred* reflects that rollovers increase severity dramatically. Farm vehicles (e.g., tractors) lack roll cages and seatbelts, leading to occupant ejection or crushing. Unpaved road edges and uneven terrain in rural areas amplify rollover risks, resulting in spinal/head injuries or fatalities. Some previous studies also indicate that rollover is a major contributor to crash severity^58–60^. Besides, the negative sign of *Collision_with_Fixed_Object* indicates reduced severity in impacts with stationary objects (trees, poles). These are often single-vehicle, controlled-deceleration events where the object absorbs energy. Farm vehicles’ high ground clearance may prevent cabin intrusion, contrasting with multi-vehicle collisions involving rotational forces.

According to the results, the positive sign of *Accident_0000_to_0600* highlights elevated severity at night (00:00–06:00). Darkness reduces visibility, while fatigue and speeding on empty roads delay evasive actions. In developing regions, limited street lighting and longer emergency response times further increase mortality. Recent research by other authors has also examined the impact of environmental variables, such as lighting, on accident severity^61,62^. Furthermore, as shown in Table 4, the negative sign of *Truck_Involved* suggests lower severity when trucks are involved. Trucks’ robust frames absorb collision energy, protecting their occupants. Farm vehicles striking trucks often experience “underride” (deceleration without cabin compromise), reducing injury risk versus head-on crashes with smaller vehicles.

On the other hand, the positive sign of *Motorcycle_Involved* signifies sharply increased severity with motorcycles. Unprotected riders suffer direct trauma when colliding with heavy farm vehicles. In developing countries, low helmet compliance turns even low-speed impacts into fatal head injuries. Numerous studies have considered the impact of motorcycles’ Presence on traffic flow and road safety^63,64^. According to the results, the positive sign of *Single_Lane_Road* indicates higher severity on single-lane roads. These narrow routes encourage risky overtaking, causing head-on collisions at combined high speeds. Limited shoulders also increase rollover risks after avoidance maneuvers.The positive role of narrow and single-lane roads on the severity of crashes has also been noted and confirmed by researchers in recent years^65–67^. As illustrated in Table 4, the negative sign of *Front_to_Rear_Collision* shows reduced severity in rear-end collisions. The striking vehicle’s crumple zone absorbs energy, while speed differentials are often low in rural traffic. Farm vehicles (struck rear) experience forward acceleration, which is biomechanically less damaging than lateral impacts.

According to the results, when a front-to-rear collision occurs and the farm vehicle is at fault (*Front_to_Rear_Collision: Farm_Vehicle_at_Fault*), injury severity decreases. This counterintuitive outcome arises because farm vehicles (e.g., tractors) involved in rear-end collisions are typically the striking vehicle due to their slow speed and high mass. At low speeds, their momentum dominates impacts, causing controlled deceleration that minimizes cabin deformation. Farm vehicle occupants experience reduced force transmission due to rigid frames and high seating positions, while struck vehicles (e.g., passenger cars) bear the brunt of energy absorption. However, this effect assumes low-speed scenarios common in rural settings; high-speed collisions may invert this pattern. While the RPOL model captures this parametric effect through random heterogeneity in fault attribution, the MC-SVM uncovers underlying non-linearities via its RBF kernel, revealing threshold dynamics where severity remains suppressed until speed differentials surpass around 40 km/h—beyond which the probability of injury escalates non-monotonically due to unstable load shifts and energy dissipation thresholds in FEV structures, as evidenced by permutation importance scores assigning 0.15 to this interaction term.

Results shows that the front-to-rear collisions on straight roads (*Front_to_Rear_Collision: Straight_Road*) increase severity. Straight roads enable higher travel speeds, amplifying kinetic energy in rear-end crashes. When a fast-moving vehicle strikes a slow farm vehicle (e.g., tractor hauling crops), the speed differential exceeds 50–70 km/h in developing countries due to poor visibility (e.g., absent reflectors). The farm vehicle’s lack of crash-absorbing structures—combined with unstable loads (e.g., grain, tools)—triggers secondary impacts: whiplash, cargo projectile injuries, or post-collision rollovers. Straight roads also reduce driver alertness, delaying evasive action. Complementing RPOL’s linear approximations, MC-SVM’s partial dependence analysis for this scenario yields a sigmoid-like curve in fatal probability, starting flat below 30 km/h differentials but rising steeply (from 0.12 to 0.68) above 50 km/h on straight alignments—illustrating non-linear amplification from reduced visual cues and sustained momentum, with feature interactions contributing 0.22 to overall model variance.

## Results and discussion

This study explored the injury severity of crashes involving Farm Equipment Vehicles (FEVs) on interurban and rural roads in a developing country context by employing two complementary modeling approaches: the Random Parameters Ordered Logit (RPOL) model and the Multi-Class Support Vector Machine (MC-SVM). The primary aims were to (1) identify critical factors influencing crash severity, (2) capture both observed and unobserved heterogeneity in those factors, and (3) determine the more accurate predictive model for crash severity classification. The findings revealed that both RPOL and MC-SVM models achieved reasonably strong predictive performance. However, based on the Area Under the Curve (AUC) metrics across severity levels, the MC-SVM outperformed RPOL, particularly in the classification of injury and fatal outcomes. MC-SVM achieved AUC values of 0.729 (PDO), 0.740 (Injury), and 0.721 (Fatal), while the RPOL model yielded 0.720, 0.657, and 0.710, respectively. This aligns with the growing literature promoting ML approaches for real-time severity prediction in ITS applications^68^. Besides, the findings reinforce the need for context-sensitive safety strategies and demonstrate the complementary value of interpretability (via RPOL) and predictive performance (via MC-SVM), consistent with recent methodological discussions in crash-severity modeling^69,70^.

The RPOL model provided important insights into the heterogeneous effects of crash-related factors. Key contributors to increased severity included motorcycle involvement, nighttime crashes (00:00–06:00), vehicle overturns, and technical defects. The significant effect of overturning is supported by evidence showing the heightened vulnerability of agricultural and off-road vehicles to rollovers^71,72^. Conversely, reversing maneuvers and rear-end collisions were associated with lower severity. Importantly, the RPOL model captured heterogeneity in the effects of front-to-back collisions depending on whether the FEV was at fault or if the crash occurred on a straight road. Similar context-dependent heterogeneity has been emphasized in recent rural-safety modeling studies^73,74^, underscoring the need to account for roadway geometry, lighting conditions, and crash configuration rather than relying on aggregate averages. This demonstrates the importance of contextualizing crash risk based on both crash configuration and road environment, which is often overlooked in conventional models.

Beyond main effects, several two-way interaction terms proved statistically significant in the RPOL model and revealed important heterogeneous patterns. The interaction Front-to-Rear Collision × Farm Vehicle at Fault produced a negative coefficient with a normally distributed random parameter, indicating that when the slow-moving FEV is the striking vehicle, severity is generally lower, but this protective effect varies substantially across crashes and disappears in approximately 6% of cases. Conversely, the interaction Front-to-Rear Collision × Straight Road yielded a positive and significant random parameter, showing that rear-end crashes on straight alignments dramatically increase severity—likely due to higher approach speeds and larger speed differentials. A similar amplifying effect was observed for Overturn × Straight Road, confirming that overturns on straight sections are far more dangerous because tractors can reach higher speeds before losing control. Nighttime × Single-Lane Road also significantly raised severity, with heterogeneity suggesting that the risk is particularly pronounced on narrow rural roads lacking lighting or markings. These interactions highlight that the same crash type (e.g., rear-end or overturn) can have opposite severity outcomes depending on road geometry and fault attribution—a nuance that would be missed in conventional fixed-parameter models. The MC-SVM analysis complements these findings: permutation importance ranked several interaction-derived features (e.g., speed differential proxies combined with alignment) among the top 10 contributors, and partial dependence plots displayed clear non-linear thresholds (e.g., severity escalates sharply once the speed differential exceeds ~ 45 km/h on straight roads). Together, these results underscore the critical importance of explicitly modeling interaction effects and heterogeneity when developing evidence-based safety countermeasures for FEVs in developing countries.

From a policy perspective, several interventions are recommended to enhance rural road safety for FEV operations. These include (1) renewal or retirement of old and mechanically deficient FEVs, (2) restrictions on nighttime operation of such vehicles, (3) targeted enforcement and awareness campaigns addressing motorcycle-FEV interactions, and (4) infrastructure improvements, particularly on single-lane and straight rural roads where severity risks are elevated. These recommendations align with evidence from rural-crash research in developing contexts^73^ as well as farm-vehicle safety studies emphasizing equipment maintenance and visibility enhancement^75^. Additionally, equipping FEVs with rollover protection systems, reflective markings, and lighting enhancements may mitigate the effects of low-visibility conditions and improve detection by other drivers.

Despite its contributions, this study is not without limitations. The data were limited to reported crashes on interurban and rural roads in Iran, potentially omitting unreported or minor incidents. Moreover, variables such as driver behavior, weather conditions, or precise vehicle configurations may have been underrepresented due to data availability. Future research could benefit from integrating more granular exposure data, vehicle telematics, or naturalistic driving datasets to better quantify causal pathways. Comparative studies across other developing countries would also validate the generalizability of the findings.

Overall, this study contributes to the growing body of literature by combining statistical and machine learning models to both understand and predict injury severity in FEV-involved crashes. The results underscore the need for context-sensitive safety policies and the value of interpretable, heterogeneity-aware modeling frameworks in traffic safety analysis.

## Data Availability

The datasets analyzed during the current study are not publicly available due to confidentiality but are available from the corresponding author on reasonable request.
